# The Use of Polysaccharide Matrices as a Basis for the Formation of Tellurium Nanoparticles with Different Morphologies

**DOI:** 10.3390/polym16111482

**Published:** 2024-05-23

**Authors:** Marina Zvereva

**Affiliations:** A.E. Favorsky Irkutsk Institute of Chemistry SB RAS, 1 Favorsky Street, 664033 Irkutsk, Russia; mlesnichaya@mail.ru

**Keywords:** polysaccharides, nanocomposites, tellurium, synthesis, galactose, base-reducing system, telluride-anions, nanorods

## Abstract

The widening of possible areas of practical uses for zero-valent tellurium nanoparticles (Te^0^NPs) from biomedicine to optoelectronic and thermoelectric applications determines the actuality of the development of simple and affordable methods for their preparation. Among the existing variety of approaches to the synthesis of Te^0^NPs, special attention should be paid to chemical methods, and especially to “green” approaches, which are based on the use of precursors of tellurium in their powder bulk form and natural galactose-containing polysaccharides—arabinogalactan (Ar-Gal), galactomannan—(GM-dP) and κ-carrageenan (κ-CG) acting as ligands stabilizing the surface of the Te^0^NPs. The use of basic-reduction system “N_2_H_4_ H_2_O-NaOH” for preliminary activation of bulk-Te and Ar-Gal, GM-dP and κ-CG allowed us to obtain in aqueous medium a number of stable nanocomposites consisting of Te^0^NPs stabilized by the polysaccharides’ macromolecules. By varying the precursor ratio, different morphologies of nanoparticles were obtained, ranging from spheres at a polysaccharide/Te ratio of 100:1 to rice-like at a 10:1 ratio. The type (branched, combed, or linear sulfated) of polysaccharide and its molecular weight value determined the size of the nanoparticles. Thus, the galactose-containing polysaccharides that were selected for this study may be promising renewable materials for the production of water-soluble Te^0^NPs with different morphology on this basis.

## 1. Introduction

Together with O, S, Se, and Po, tellurium is a typical representative of chalcogens. It is a rare low-toxicity metalloid, the content of which in the earth’s crust does not exceed 1 × 10^−6^ % wt. [1]. In its solid state, tellurium has several allotropic modifications, but in practice, hexagonal silver-gray tellurium with a metallic appearance is the most abundant. This structure consists of parallel spiral chains located at the angles and in the center of the elementary hexagonal cell [2]. The result of this crystal lattice structure of hexagonal Te is its pronounced chirality, and, accordingly, its ability to form a crystal lattice along certain faces and directions, forming one-dimensional (1D) nanostructures [3]. Additionally, due to van der Waals (or, according to other sources, coordination-covalent) interactions, several such Te chains can form stable two-dimensional (2D) structures, resembling a film with a thickness of only one layer [4,5,6,7]. The zone structure of Te determines its pronounced semiconductor properties with p-type conductivity at room temperature, as well as its ability to exhibit nonlinear optical response, ultrafast electronic excitation, photoconductivity, and pronounced thermoelectric properties, including a high Seebeck coefficient. However, the low value of the forbidden band width of bulk Te (0.33–0.35 eV) underlies a significant increase in leakage current in prototype transistors based on it. Conversion of Te into its nanoscale state, due to the effect of quantum confinement, can significantly increase the electronic gap value. Examples of successful preparation of anisotropic (nanowires, nanofibers) nanostructures based on Te with an Eg value greater than 1 eV are provided in [8,9,10]. Due to their excellent stability and ability, under van der Waals forces, to form a material as thin as a single layer, 2D Te-based nanomaterials exhibit an increased transmission gap of up to 1.26 eV, making them popular materials for creating photodetectors for broadband detection [11]. Moreover, nanomaterials based on zero-valent Te (Te^0^NPs) show promising applications in biomedicine, particularly as antimicrobial, anti-inflammatory, and antiradical agents [12,13,14]. For instance, Te^0^NPs have demonstrated significant bactericidal effects against methicillin-resistant *Staphylococcus aureus* [15], *Pseudomonas aeruginosa*, *Salmonella typhi*, *Klebsiella pneumonia* [13], *E. coli*, *P. aeruginosa*, *S. typhi*, *MRSA*, *C. albicans*, and *C. dubliniensis*, among other pathogens. In addition, we found a pronounced antimicrobial effect against the bacterial phytopathogen *Clavibacter sepedonicus* in our preliminary studies on the production of Ar-Gal-based Te^0^NPs using tellurium oxide as a source for nanoparticles [16].

It should also be noted that the zero-valent state of Te is its least toxic form compared to its previously utilized tellurite and tellurate forms [17]. Additionally, the nanoscale size of zero-valent Te enhances its bioavailability and solubility, particularly when the surface of Te^0^NPs is coated with hydrophilic ligands. This coating ensures the preservation of its complete profile of biological activity, similar to its more toxic water-soluble forms. The challenge of kinetic and aggregative stability of Te^0^NPs is addressed through the use of surface-passivating ligands, preventing particle aggregation and maintaining their nanoscale state. This passivation of the nanoparticle surface occurs during their synthesis, following the formation of nuclei of a new phase, and the subsequent growth and maturation of nanoparticles.

Over the past decade, significant advancements have been made in developing new strategies for synthesizing various Te^0^NPs with precise control over composition, size, morphology, and properties, thereby expanding their applicability across numerous fields. Particularly noteworthy are methods utilizing natural plant extracts or bacteria for the synthesis of Te^0^NPs, which have gained widespread attention [18,19,20,21,22,23,24]. The simplicity and availability of chemical liquid-phase approaches to nanoparticle synthesis determines their extension to the synthesis of elemental Te^0^NPs, including those of various morphologies (nanospheres, nanotubes, nanofibers, nanorods, etc.). Among the most commonly used techniques for the synthesis of Te^0^NPs are solvo- and hydrothermal methods and polyol synthesis, as well as a combination of the conditions of the above methods with exposure to ultraviolet or visible radiation, ultrasound, microwave, and laser radiation [6,25,26].

The most actively used approaches to the production of Te^0^NPs are based on the recovery of Te from its precursors. The precursors are low molecular weight Te-containing compounds (H_2_TeO_4_·2H_2_O, Na_2_TeO_3_, TeO_2_, diethyldithiocarbamate of Te) in which the reduction itself is provided either by introduction of active reducing agents (ascorbic acid, sodium borohydride, formamide, hydrazine hydrate) or by physically-induced (light, temperature, microwave or laser radiation, etc.) oxidation of solvent molecules (e.g., water in hydrothermal synthesis, alcohols in solvothermal synthesis, ethylene glycol in polyol synthesis) [6,27]. In addition, there are a number of studies describing the synthesis of Te^0^NPs using alkali metal tellurides as their source. In these cases, the synthesis of nanoparticles is based on the oxidation of Te anions to the zero-valent state, including with the involvement of various oxidizing agents [28]. Elemental powder Te can also be used for the synthesis of Te^0^NPs only after its preliminary activation (e.g., in the systems NaBH_4_-NH_4_OH or NaOH-N_2_H_4_-H_2_O) [29,30].

Nevertheless, despite the variety of approaches, the synthesis of stable Te^0^NPs in a simple and environmentally friendly way still remains a challenge. Existing techniques cannot always satisfy the basic principles of green chemistry due to the need for toxic flammable solvents, precursors, expensive apparatus design, high temperatures, or exotic reagents. Meanwhile, biogenic synthesis methods using plant extracts or culture fluids of bacteria and fungi determine a strict dependence of the characteristics of the obtained nanomaterials on the concentration of components included in their composition. This, in turn, significantly complicates the standardization of nanomaterials and makes large-scale industrial production of nanomaterials based on them practically impossible.

At the same time, another natural raw material—polysaccharides—is easily subjected to standardization primarily by such an important parameter as monomeric composition and molecular weight. Their wide distribution and renewable of natural sources makes their resource practically unlimited and economically accessible [31,32]. To date, a number of nanoparticles of different nature (noble metals, metal oxides, metal chalcogenides, and elemental chalcogenides) have been successfully prepared on the basis of polysaccharides [33,34]. The nanomaterials obtained on their basis combined hydrophilicity and the biological and rheological properties of polysaccharide-stabilizing ligand and biological and physicochemical properties of nanoparticles themselves. Despite such an active use of polysaccharides for the synthesis of nanoparticles, there are not many examples of successful production of Te^0^NPs on this basis. Thus, in [35,36,37], the authors describe the synthesis of Te^0^NPs on chitosan. In [38], Te nanofibers were obtained using the stabilizing ability of starch. Taking into account the great potential for practical use of Te^0^NPs, as well as the availability and pronounced nanostabilizing ability of polysaccharides, their use for the synthesis of biocompatible, water-soluble Te^0^NPs is very attractive, especially from the point of view of the basic principles of green chemistry. Previously, water-soluble galactose-containing polysaccharides such as arabinogalactan (Ar-Gal), galactomannan (GM), and κ-carrageenan (κ-CG) have been used by us to synthesize nanoparticles of silver, gold, iron oxides, elemental sulfur, and selenium, as well as a number of metal sulfides, selenides, and tellurides [33]. The obtained nanomaterials demonstrated high hydrophilicity, long-term aggregative stability, and low toxicity, as well as the presence of a number of biological (antimicrobial activity, antioxidant activity, antifungal activity, antianemic activity) and physical properties (luminescence, magnetic properties, optical activity). Obtaining Te^0^NPs on the basis of these polysaccharides in the future could be an effective and safe approach to the synthesis of Te-containing nanomaterials combining the whole complex of biological and physical properties and Te nanophase and hydrophilicity and biological properties of polysaccharides stabilizing their surface.

This work is aimed at the development of a synthetic approach to the preparation of stable water-soluble nanoparticles of zero-valent from natural nanostabilizing galactose-containing polysaccharides, arabinogalactan, galactomannan and κ-carrageenan. The dependence of structural and nanomorphological characteristics of the nanomaterials on the type of stabilizing ligand has been studied in detail.

## 2. Materials and Methods

### 2.1. Materials

In this work, we used Ar-Gal isolated from Siberian larch (*Larix Sibirica*). It is a white amorphous powder, highly soluble in water. The isolation and purification of Ar-Gal was carried out according to the protocol described in detail in [39]. Found, %: C-41.9, H-7.4, O-50.7. IR spectra (KBr, ν, cm^−1^): 3422 (OH), 2920 (C-H), 1642 (HH), 1378–1077 (C-C, C-O), 876 and 776 (C-H bonds of pyranose rings). GM LBG mark (MM 2300 kDa) and κ-CG WR-78 mark (MM 1800 kDa) (CP Kelco, Lille Skensved, Denmark) were used without further purification. To synthesize nanocomposites, samples of polysaccharides that underwent preliminary partial alkaline depolymerization were used according to the method described in detail in [40] with minor modifications. It was possible to obtain samples with improved water solubility and reduced viscosity of solutions compared to samples of depolymerized GM (GM-dP) and κ-CG (κ-CG-dP) according to the process presented in [40].

All other reagents, including Te^0^ powder (Alfa Aesar, Karlsruhe, Germany), NaOH, HCl, N_2_H_4_·H_2_O (64% solution), and ethanol (all Reachim), were used without further purification.

### 2.2. Methods

Microphotographs of the samples were taken following standard procedure on a Tecnai G2 20F S-TWIN FEI transmission electron microscope (FEI Europe B.V., Eindhoven, The Netherlands). Aqueous solutions of composites with a concentration of 1.6 mg mL^−1^ were used for microscopy. Next, a drop of the resulting nanocomposite solution was placed on a substrate grid (Formvar 200 Mesh, Copper, 50 p), followed by air-drying. The size distribution of the nanoparticles was determined by statistical processing of microphotographs with use IP Win45 software package (Version 4.5.0.29).

The elemental analysis was carried out by X-ray energy dispersive microanalysis using a Hitachi TM 3000 electron scanning microscope (Angstrom Scientific Inc., Ramsey, NJ, USA) with an SDD XFlash 430-4 X-ray detector and a Thermo Scientific Flash 2000 CHNS analyzer (Thermo Fisher Scientific, Waltham, MA, USA).

The hydrodynamic radii (*R_h_*) of polysaccharide-stabilized Te^0^NPs were determined by dynamic light scattering (DLS) on a Photocor Compact-Z correlation spectrometer (Photocor, Moscow, Russia) (the light source was a 20 mW thermo stabilized semiconductor laser with a wavelength of λ = 638 nm). The correlation function was analyzed using Dynals DLS data processing software (Version 2.0). The solutions for analysis were prepared by dissolving, for at least twelve hours, a 15 mg sample in 30 mL of distilled water, which was pre-filtered through a syringe filter for a finished solution with a 0.5 gL^−1^ concentration. The obtained solution was then purified again by filtration through a syringe filter (0.22 µm) [41]. The time for each measurement was at least 200 s. The scattering angle was 90°. The measurement was taken in triplicate. The polyelectrolyte velocity ν under external electric field E was measured. The electrophoretic mobility μE = ν/E was converted into the Zeta potential (the potential of the electrical double-layer at the surface of hydrodynamic shear) by the Smoluchovsky equation, μE = εε_o_ζ/ηs, where ε is the solvent dielectric permittivity, ε_o_ presents vacuum dielectric permittivity, and ζ is ζ-potential. Each measurement was performed 3 times, and the results were averaged.

Optical absorption spectra of 0.2% aqueous solutions of the nanocomposites and the original polysaccharides (Ar-Gal, GM-dP and κ-CG-dP) were taken on a Perkin Elmer Lambda 35 spectrophotometer (PerkinElmer, Waltham, MA, USA) in a 1 cm quartz cuvette using distilled water as a reference solution.

FT-IR spectra were recorded by a FT-IR (RAM II) Bruker Vertex 70 spectrometer (Bruker, Ettlingen, Germany) in KBr pellets in the range of 4000–400 cm^−1^.

#### 2.2.1. Partial Alkaline Depolymerization of GM and κ-CG (General Methodology)

To a 1.6% aqueous solution of GM (or κ-CG) (120 mL), a 1 N aqueous solution of NaOH (30 mL) was slowly added under vigorous stirring at 45 °C. Afterwards, the temperature was increased to 90 °C, and the mixture was thermostated for 180 min upon constant stirring. After this time, the reaction mixture was neutralized with an equimolar amount of HCl, followed by dialysis against distilled water for 24 h. The depolymerized polysaccharide samples were isolated by precipitation of the dialyzed reaction medium into ethanol, separation of the precipitate on a Schott filter, and drying at room temperature and atmospheric pressure. The yields of depolymerized GM and κ-CG samples were 81 and 89%, respectively. The following were found (GM-dP), %: C 46.21; H 6.25; O 47.54. IR spectra (KBr, ν, cm^−1^): 3433 (OH), 2924, 2877 (C-H), 1642 (HOH), 1200–812 (C-C, C-O), 870 and 812 (β-glycosidic bonds of pyranose residues). The following were found (κ-CG-dP), %: C 31.93; H 5.80; O 49.04; S 6.24; K 3.37; Na 3.62. IR spectra (KBr, ν, cm^−1^): 3437, (OH), 2996, 2921, 2854 (C-H), 1320–1070 (C-O), 933, 757 (C-H bonds of pyranose cycles), 1638 (HOH), 1255, 846 (C-O-S sulfoether groups).

#### 2.2.2. Generation of Telluride Anions from Tellurium Powder

A sample of NaOH (0.1 g), N_2_H_4_·H_2_O (64%, 0.1 mL) and distilled water (0.4 mL) was placed in a 50 mL three-neck flask equipped with a thermometer and a reflux condenser. The mixture was stirred until NaOH was completely dissolved. Then the reaction mixture was blown with argon, and the temperature was increased to 75 °C. Under constant stirring and argon flow, powdered tellurium (0.159 g) was added, and the reaction mixture continued to be stirred under the specified conditions for another 30 min until complete dissolution of tellurium powder. The color of the reaction medium changed from colorless to red-violet, indicating the formation of telluride and polytelluride anions. The obtained reaction mixture was further used for the synthesis of elemental tellurium nanocomposites based on natural polysaccharide matrices Ar-Gal, GM-dP, and κ-CG-dP.

#### 2.2.3. Synthesis of Polysaccharide-Capped Tellurium Nanoparticles: General Procedure

The nanocomposites were synthesized after the preliminary generation of telluride anions from commercial tellurium powder in the presence of base-reducing system “hydrazine hydrate-alkali” (see previous section). An aliquot (*V* = 10–90 μL) of the reaction medium with Te^2−^ anions was added to a reactor containing 30 mL of a 1% polysaccharide solution (Ar-Gal/GM-dP/κ-CG-dP), and the reaction mixture was stirred at room temperature for 25 min. Isolation of nanocomposites and their purification from impurities was carried out by precipitation of the reaction mixture in a 4-fold excess ethanol, followed by separation of the precipitate on a Schott filter, repeated washing with ethanol, and drying in a vacuum oven. The yields were 68–83%. The obtained composites are water-soluble gray-brown or gray-blue powders.

The following were found (Ar-Gal-Te^0^NPs-0.5), %: C—38.11, H—6.76, Te—0.50, O—54.63; (Ar-Gal-Te^0^NPs-2.6), %: C—40.99, H—6.26, Te—2.63, O—50.12; (Ar-Gal-Te^0^NPs-5.8), %: C 40.05, H—6.45, Te—5.83, O—47.67; (GM-dP-Te^0^NPs-0.6), %: C 39.72, H—6.70, Te—0.60, O—52.98; (GM-dP-Te^0^NPs-3.1), %: C 42.23, H—6.52, Te—3.13, O—48.12; (GM-dP-Te^0^NPs-8.1), %: C 38.67, H—6.03, Te—8.12, O—47.18; (κ-CG-dP-Te^0^NPs-0.8), %: C 32.00, H—6.65, Te—0.80, K—3.2, Na—3.9, S—7.9, O—45.55; (κ-CG-dP-Te^0^NPs-3.5), %: C 31.42, H—6.34, Te—3.51, K—3.1, Na—3.8, S—7.6, O—44.23; (κ-CG-dP-Te^0^NPs-6.2), %: C 30.03, H—5.59, Te—6.20, K—3.0, Na—3.4, S—7.1, O—44.71.

## 3. Results

### 3.1. Structure of the Polysaccharide Macromolecules Ar-Gal, dP-GM, and dP-κ-CG

The stabilizing ability of natural galactose-containing polysaccharides (Ar-Gal, dP-GM and κ-CG-dP) was employed in the synthesis of tellurium nanoparticles. These polysaccharides are reserve nutrient metabolites of plant organisms and algae [42,43,44]. The content of Ar-Gal in Siberian larch wood can be up to 35% of the dry weight of wood depending on the characteristics of the raw materials (age of wood and location and time of collection) [42]. The most important natural sources of GM are guar gum and carob gum obtained from the endosperm of their seeds. The GM content in these raw materials reaches 75–85% [43]. Meanwhile, the main (up to 90%) source of κ-CG is red seaweeds of the *Rhodophyceae* family. The content of κ-CG in them, depending on the stage of vegetation and localization of the place of raw material collection, reaches 38–45% of the dry weight of algae [40,44]. At the same time, the renewability of their natural sources creates prerequisites for their use in various chemical applications, including the production of nanomaterials.

**Figure 1:**
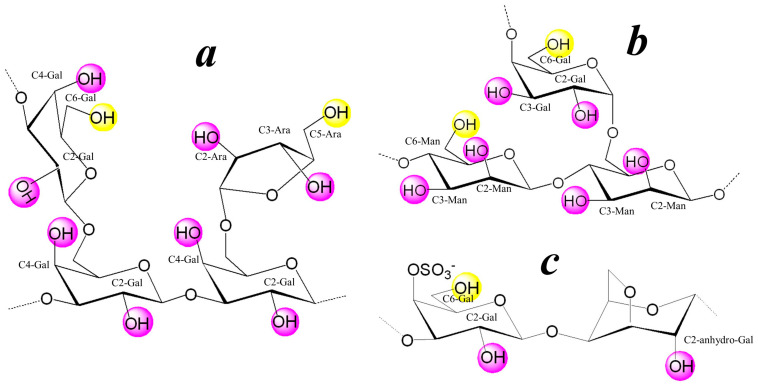
Macromolecule fragments of natural heteropolysaccharides Ar-Gal (**a**), GM (**b**) and κ-CG (**c**).

The data from elemental analysis, FT-IR, and ^13^C NMR spectroscopy show that AG extracted from Siberian larch (*Larix Sibirica*) is a branched heteropolysaccharide. The main chain of its macromolecule consists of 1→3 linked β-D-galactopyranose residues, most of which have side branches at C-6 represented by β-D-galactopyranose and β-L-arabinofuranose residues. The ratio of galactose to arabinose is 9:1 (Figure 1a). The molecular weight (Mw) of the Ar-Gal used in this work is 43 kDa [45]. GM is a comb heteropolysaccharide, the carbohydrate chain of which consists of β-D-mannopyranoside residues linked by 1→4 glycosidic bonds (Figure 1b). The side branches of GM macromolecule contain single α-D-galactopyranose residues, which are unevenly distributed along the main chain linked to the mannopyranoside chain through 1→6 glycosidic bonds [40]. The κ-CG macromolecule is built from regularly alternating residues of 3-O-substituted β-D-galactopyranose with a sulfo group at position 4 and 4-O-substituted 3,6-anhydro-α-D-galactopyranose [40] (Figure 1c). All of the above polysaccharides are natural renewable raw materials widely used in the food and pharmaceutical industries [46].

At the same time, these polysaccharides find application not only due to their unique hydrodynamic characteristics, in particular high moisture-retaining capacity and gel-forming properties, but to non-toxicity and biocompatibility, which makes their use in the food industry safe for health [46]. Particular attention is paid to the membranotropic activity inherent in galactose-containing polysaccharides such as Ar-Gal, GM and κ-CG [47]. This activity is associated with the presence of galactopyranose residues in their macromolecules. Given that galactopyranose has an affinity to asialoglycoprotein receptors of cell membranes [48], such polysaccharides can be considered a very promising platform for the design of drugs for targeted delivery of the active substance to target cells [49,50,51]. These polysaccharides differ from each other in terms of chemical composition, structure, and molecular weight, and, accordingly, their biological and rheological properties. In the long run, this allows one to predict biological activity or rheological characteristics of nanocomposites synthesized on the basis of these polysaccharides. Moreover, a wide range of biological activities of natural polysaccharides, along with their valuable molecular weight characteristics, have stimulated the active research of this group of natural compounds over several decades. The presence of polar carbonyl and primary (highlighted in yellow in Figure 1) and secondary (highlighted in purple in Figure 1) hydroxyl groups in the composition of Ar-Gal, GM, and κ-CG macromolecules determines their use as functional reagents for fine organic synthesis, including nanocomposite materials with a complex of valuable physicochemical and biological properties [52,53]. The physical–chemical and biological properties of GM and κ-CG are determined by their monomer composition, the type of functional groups contained in the macromolecules, and their molecular weight characteristics, the latter parameter being easily varied. The viscosity–average molecular weight (M_w_) of GM and κ-CG is quite high (2300 and 1800 kDa, respectively), which leads to the limited solubility (up to 10 mg/mL) of these polysaccharides in water, high viscosity of their aqueous solutions, and, as a consequence, low reactivity and low reproducibility of syntheses. The water solubility of these high molecular weight polysaccharides and their reactivity can be improved by a controlled decrease in the degree of polymerization, i.e., molecular weight of GM and κ-CG macromolecules, the functional features of the polysaccharides being preserved.

In the present work, the samples of the starting GM and κ-CG underwent partial alkaline depolymerization to afford GM-dP and κ-CG-dP species with a significantly reduced molecular weight (220 kDa and 180 kDa, respectively). The obtained compounds had good water solubility, and their FT-IR spectra corresponded to those of the original polysaccharides, thus confirming the preservation of the carbohydrate structure. The elemental analysis of the prepared GM-dP and κ-CG-dP samples and a brief description of their FT-IR spectra are given in the experimental section.

### 3.2. Synthesis of the Polysaccharides-Stabilized Te^0^NPs

Water-soluble, aggregate-stable nanocomposites consisting of polysaccharide-stabilized Te^0^NPs in the amount of 0.5–8.1% were synthesized in aqueous medium using powder tellurium as a source of highly reactive telluride anions, Te^2−^, previously generated in the base-reducing system “hydrazine hydrate-NaOH”. Previously, this system for the activation of elemental chalcogenes was described in detail in [54], devoted to the synthesis of organochalcogenic compounds. Owing to its chemical inertness, elemental bulk Te powder is not able to act as a direct source of tellurium in the synthesis of nanoparticles in the “bottom-up” approach we use. However, its transfer to Te^2−^ via reduction in the N_2_H_4_·H_2_O-NaOH system significantly increased its reactivity. The generated anions in the structure of sodium telluride are ultimately oxidized to zero-valent tellurium in an aqueous solution of polysaccharide matrices Ar-Gal, GM-dP and κ-CG-dP. In this case, the dissolution of elemental tellurium in the hydrazine hydrate-alkali system and the formation of highly reactive telluride anions is described by the Equation (1):

2Te + 4NaOH + N_2_H_4_·H_2_O = 2Na_2_Te + N_2_ + 5H_2_O (1)

The molar ratio of Te/NaOH/N_2_H_4_-H_2_O ensuring reliable and complete dissolution of tellurium is 2:1:1. The generated Te^2−^ anions were stable only at the moment of their preparation in the reaction medium. Upon cooling or in the absence of inert conditions, they were oxidized to tellurium atoms, followed by aggregation and formation of a coarse precipitate of gray tellurium. Therefore, telluride anions were synthesized immediately before preparation of nanocomposites. The quantitative content of tellurium in the polysaccharide-based nanocomposites was varied by changing the polysaccharide/Te^2−^ ratio from 100:1 to 10:1. The introduction of an aliquot of the reaction mixture containing Te^2−^ into the aqueous solution of polysaccharide matrices Ar-Gal, GM-dP and κ-CG-dP is accompanied by the development of two processes—the first of which is the hydrolysis of sodium telluride, synthesized previously from powdered tellurium and being a source of telluride anions. In this case, due to the dibasic acid telluric acid, hydrolysis of sodium telluride anion proceeds in two steps, i.e., with the formation of NaHTe and its subsequent theoretically possible hydrolysis to H_2_Te and its removal from the reaction medium due to its low solubility according to Equations (2) and (3). However, the low degree of electrolytic dissociation of NaHTe causes its “delay” in the reaction medium, and consequently the possibility of redox reaction, which results in the oxidation of hydrotelluride anions to the zero-valent state of Te^0^ described by Equation (4). Nevertheless, a part of NaHTe, especially in the case of introduction of a large volume of reaction medium containing Te^2−^ undergoes hydrolysis with the release of H_2_Te identified by the appearance of a characteristic smell of radish and a decrease in the amount of tellurium in the obtained samples compared to the theoretically expected amount.

Na_2_Te + HOH = NaHTe + NaOH (2)

NaHTe + HOH = H_2_Te + NaOH (3)

2NaHTe + O_2_ = 2Te^0^ + 2NaOH (4)

The formed Te^0^ atoms accumulating in the reaction medium under conditions of intensive stirring collide with each other and form nuclei of a new solid phase when the critical concentration is reached. In this case, the amount of free tellurium atoms in the reaction medium after the onset of the nucleation stage falls sharply and the formation of new nuclei becomes impossible. Meanwhile, the formed nuclei of the solid phase complete their crystal lattices due to the permanent attachment of “free” tellurium atoms or “released” due to the re-dissolution of small unstable particles of tellurium atoms to the surface of growing nanoparticles. In this case, the solubility of particles increases significantly with decreases in their size due to the increase in the specific surface area interacting with the dispersion medium. The growth of particles is limited by the diffusion of tellurium atoms to their surface and the number of tellurium atoms capable of completing the crystal lattice of the growing nanoparticle in the volume of the reaction medium. The growth and maturation of Te^0^NPs in the process of synthesis of nanocomposites is accompanied by a drop or complete disappearance of Te^0^ atoms and a decrease in the solubility of nanoparticles due to an increase in their size; that is, further growth of nanoparticles due to the attachment of reduced tellurium atoms to the surface of growing nanoparticles by the LaMer mechanism becomes impossible [55].

In this case, the telluride anions present in the reaction medium are able to complete the crystal lattice of the formed Te^0^ nanoparticles, adsorbing on their surface and determining the charge of the nanoparticle surface; i.e., they act as potential-determining ions. Meanwhile, Na^+^ and OH^−^ ions also present in the reaction medium (due to their high sorption capacity) are counterions and can be part of the double electric layer of Te^0^NPs, limiting their coalescence, that is, provide in the complex of their electrostatic stabilization due to repulsion of negatively charged particles of the same name from each other. Adsorption of Ar-Gal, GM-dP and κ-CG-dP macromolecules present in the reaction medium on the surface of Te^0^NPs increases their aggregative stability by the mechanism of steric stabilization, due to the screening of a number of interparticle interactions and the realization of the structural and mechanical stability factor of the resulting lyophobic sols (Te^0^NPs) [56].

Realization of electrostatic stability factor of Te^0^ nanoparticles in this case is possible only for a short time (until all available Na_2_Te molecules are not subjected to hydrolysis with the formation of NaHTe or oxidation to Te^0^) during the synthesis of nanoparticles or absolutely excluded after stopping the synthesis and separation of polysaccharide-Te^0^ nanocomposites from the reaction medium due to the removal of all unreacted substances or reaction by-products in the process of isolation and purification. Meanwhile, it is the passivation of the surface of Te^0^NPs by Ar-Gal, GM-dP and κ-CG-dP macromolecules that provides their long-term (over one month) aggregative and kinetic stability [57,58].

### 3.3. Characterization of Structure and Nanomorphology of Polysaccharide-Stabilized Tellurium Nanoparticles

#### 3.3.1. Determination of Nanoparticle Morphology and Characterization of their Structure by TEM and HR-TEM

**Figure 2:**
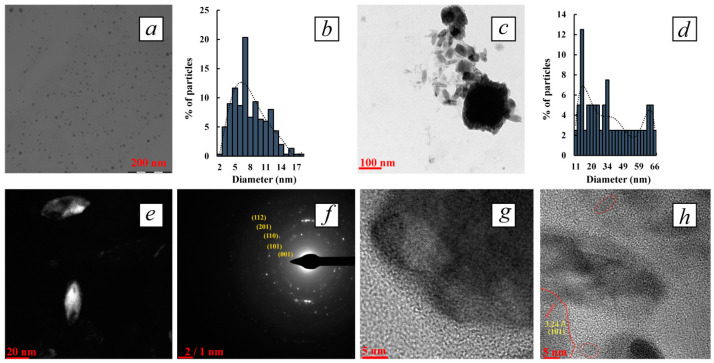
TEM micrograph (**a**) and dispersion distribution diagram (**b**) of Te^0^NPs in Ar-Gal matrix (0.5% Te); TEM micrograph (**c**) and dispersion distribution diagram (**d**) of tellurium nanoparticles in Ar-Gal matrix (5.8% Te); dark-field micrograph of rice-like Te^0^NPs (**e**), SAED (**f**) and HR-TEM images (**g**,**h**) of Te^0^NPs (5.8% Te).

**Figure 3:**
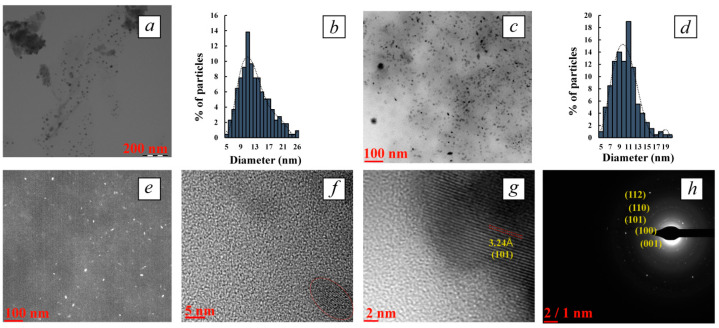
TEM micrograph (**a**) and dispersion distribution diagram (**b**) of Te^0^NPs in GM-dP matrix (0.6% Te); TEM micrograph (**c**) and dispersion distribution diagram (**d**) of Te^0^NPs in GM-dP matrix (8.1% Te); dark-field micrograph of rice-like Te^0^NPs (**e**), HR-TEM images (**f**,**g**) and SAED of Te^0^NPs (**h**) (all 8.1% Te).

**Figure 4:**
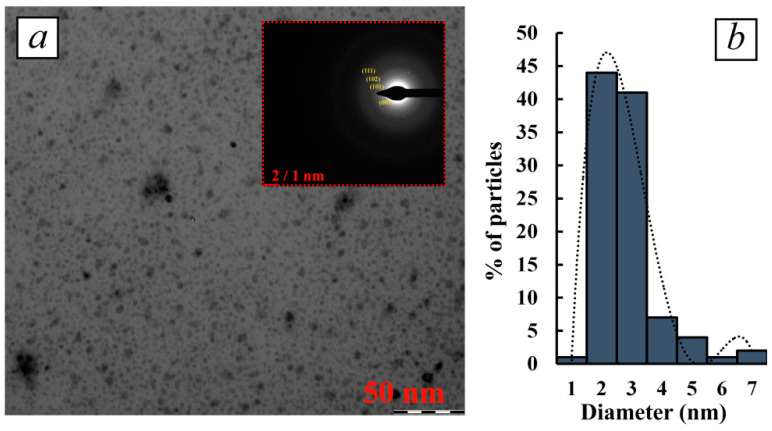
TEM micrograph (**a**) and dispersion distribution diagram (**b**) of Te^0^NPs in κ-CG-dP matrix (0.8% Te); inset SAED of Te^0^NPs.

The main nanomorphological characteristics (size, shape, average diameter, character of dispersal distribution) of the obtained tellurium-containing nanocomposites were determined using transmission electron microscopy, including high-resolution TEM (TEM and HR-TEM). It was found that the synthetic conditions and, first of all, the polysaccharide/Te^2−^ ratio, as well as the type of polysaccharide dramatically affected the nanomorphological parameters of the tellurium nanoparticles. It was established that at highest polysaccharide/Te^2−^ ratio (100:1) and with a content of the inorganic phase of 0.5–0.8%, the nanocomposites represented close to spherical particles that were distributed in Ar-Gal, GM-dP and κ-CG-dP matrices, the average size of the particles ranging from 2.8 to 12.9 nm (Figure 2, Figure 3 and Figure 4). Thus, in the Ar-Gal polysaccharide matrix with an Ar-Gal/Te^2−^ ratio of 100:1, spherical nanoparticles of 2–17 nm in size (on average 7.9 nm) were formed (Figure 2a,b). Only 36% of the particles had a size of 7–9 nm, which indicated a fairly high polydispersity of the particles in the sample. An increase in tellurium content in the composites to 5.8% (Ar-Gal/Te^2−^ ratio = 10:1) led to the formation of large close to oval particles with a longitudinal size of 11–66 nm, (on average 36.8 nm) and a length/width ratio of 2.1–3.7. The nature of the disperse distribution in this case changed from lognormal with positive symmetry to close to uniform (Figure 2c,d). The high (2.2) value of the asymmetry coefficient (*As*) of the lognormal distribution in the first case is probably due to the low symmetry of the obtained distribution with the predominance of the fraction of small particles (2–8 nm), which may be associated with the process of nanoparticle formation by the mechanism of sequential adsorption of Te^0^ atoms on the surface of growing particles. Meanwhile, close to uniform distribution with the separation of several size groups of “rice-like” Te^0^NPs in Ar-Gal-Te composites (5.8% Te) indicates the realization of another mechanism of their formation, namely by means of either coalescence, i.e., random merger of several small nanoparticles into larger ones, or oriented attachment [59,60]. These two mechanisms of nanoparticle growth are different from each other by the orientation of the crystal lattice at the grain boundaries. In coalescence, there is no special preference for the attachment of nanoparticles to each other, which results in the formation of aggregates combining several fragments of crystal lattices without a uniform orientation of their planes. Meanwhile, oriented attachment is accompanied by a general crystallographic alignment of attachment, allowing to obtain continuous crystallographic planes.

For composites based on GM-dP, a similar dependence of the shape of Te^0^NPs on the quantitative ratio of reagents in the reaction medium and the final content of inorganic phase in their composition is observed. Thus, with a GM-dP/Te^2−^ ratio of 100:1, Te^0^ particles are formed, which are identified in TEM images as close to spherical, their size varying within the range of 5–26 nm, with an average value of 13.0 nm (Figure 3a,b). As in the case of Ar-Gal-Te^0^NPs composite (0.5% Te), the size distribution of the identified nanoparticles is characterized by lognormal type with a pronounced positive symmetry and *As* value of 2.4, which in turn indicates a similar mechanism of growth of Te^0^NPs in the GM-dP matrix during synthesis. The increase in the quantitative content of tellurium in the composite on the basis of GM-dP up to 8.1% in the conditions of the ratio of precursors in the reaction medium 10:1 is accompanied primarily by the formation of Te^0^NPs whose shape deviates from spherical to “rice-like” with a length/width ratio of 1.5–2.3, with a general preservation of the size range of nanoparticles in the range of 5–19 nm (the values of longitudinal dimensions of nanoparticles are used in the calculation) and the average size of 10.3 nm (Figure 3c,d). Slight (*As* 1.1) positive asymmetry of lognormal dispersion distribution of particles also indicates the mechanism of nanoparticles growth as a result of sequential attachment of tellurium atoms formed in the reaction medium either as a result of oxidation of precursors or as a result of dissolution of unstable small-sized tellurium clusters to the surface of growing nanoparticles. The absence of evidence of massive aggregation of nanoparticles leading to a uniform character of their size distribution is probably due to the creation of conditions for the longitudinal growth of anisotropic Te^0^NPs in the matrix GM-dP, probably due to the higher viscosity of the reaction (dispersion) medium, which in turn contributes to the realization of the hydrodynamic stability factor in the formation of nanoparticles significantly slowing their diffusion and limiting the number of possible clashes of unstable nanoparticles capable of fusion [61]. In addition, under these conditions, the mechanism of nanoparticle growth through the diffusion of monomers (Te^0^ atoms) along the surface of the formed particles is also possible, which leads to a change in their shape. Thus, the faces of the particles with high energy dissolve, and the faces with low energy grow due to the released Te^0^ atoms [60].

In the case of using κ-CG-dP as a stabilizing matrix for the synthesis of Te^0^NPs, the same dependences of nanomorphological characteristics of formed nanoparticles from the ratio κ-CG-dP/Te^2−^ were observed. Thus, a significant excess of κ-CG-dP in the reaction medium compared to tellurium precursor ions under conditions of κ-CG-dP/Te^2−^ ratio 100:1 is accompanied by the formation of spherical particles whose size varies in the range of 1–7 nm, with an average value of 2.8 nm (Figure 4a,b). At the same time, 82% of all particles taken into account have a size of 2–3 nm, indicating a high monodispersity of particles formed under these conditions in the matrix κ-CG-dP. Meanwhile, the high value of *As* 3.2 and the positive asymmetry of the dispersion distribution indicate, as in the case of other polysaccharides with a similar precursor ratio, the predominance of the mechanism of sequential attachment of Te^0^ atoms to the surface of growing nanoparticles as the main one in the formation of Te^0^NPs in the κ-CG-dP polysaccharide matrix.

HR-TEM dark-field microscopy performed for all composites containing high (5.8–8.1%) amount of tellurium also visualizes high-contrast nanoparticles of elongated “rice-like” shape, which confirms their crystalline nature (Figure 2e and Figure 3e). The internal microstructure of the nanoparticles was also further investigated by HR-TEM. The obtained images confirm the presence of co-oriented lines, which obviously indicate the crystalline nature of the visualized Te^0^NPs (Figure 2g,h and Figure 3f,g). The value of interplanar distances between series of atoms in the crystal lattice is 3.24 Å, which corresponds to the (101) crystallographic plane of hexagonal tellurium (PDF #00-001-0714).

In addition, the selected area electron diffraction (SAED) pattern of all polysaccharide-stabilized Te^0^NPs shows clear and discrete over-emission points, also indicating the high crystallinity of the nanoparticles (Figure 2f, Figure 3h and Figure 4a (inset)). The electronograms of polysaccharide-stabilized Te^0^NPs is represented by symmetrical rings with randomly distributed high-intensity contrast regions with no preferred orientation, indicating the polycrystalline nature of the nanoparticles in the composites. Each of the visualized spots on the rings arises as a result of Bragg reflection from several crystals reflected at different scattering angles, which gives a continuous pattern of spots with random orientation. It was found that the lattice interface distances are 5.80, 3.86, 3.24, 2.34, 2.22, 2.08, 1.83, 1.77, 1.61, and 1.16 Å. The obtained values are very close to the values of the interplanar distance of the hexagonal lattice of the standard Te^0^ sample and correspond to its (001), (100), (101), (102), (110), (111), (201), (112), (202) crystallographic planes (ICCD card no. 00-001-0714). Comparison of crystallographic data of experimentally obtained samples of polysaccharide-stabilized Te^0^NPs with reference ones allows us to determine the presence of elemental Te^0^NPs in our nanocomposites having a crystal lattice with a primitive type of centering and hexagonal syngony (a = 4.4450 Å = b = 4.4450 Å, c = 5.9100 Å, α = β = 90°, γ = 120°).

Thus, the analysis of HR-TEM data on nanomorphology and internal structure of Te^0^NPs formed in polysaccharide matrices Ar-Gal, GM-dP and κ-CG-dP allows us to confirm the predominant influence of polysaccharide/Te^2−^ ratio in the reaction medium on these characteristics. This is confirmed by the revealed tendency to anisotropy of Te^0^NPs growth under conditions of increasing quantitative content of inorganic nanophase in the composite composition regardless of the type of polysaccharide used. Meanwhile, the size characteristics of Te^0^NPs are determined primarily by the type of polysaccharide used. Thus, the smallest average size and polydispersity were demonstrated by the Te^0^NPs formed in κ-CG-dP matrix. Meanwhile, the largest size and polydispersity were exhibited by the Te^0^NPs in Ar-Gal matrix. Probably, κ-CG-dP macromolecules, due to their polyanionic nature, are able to change the conformation of their chains in the presence of other ions (Na^+^, OH^−^), although the influence of these ions is not as dramatic as the specific influence of K^+^ ions, which can lead to a rapid transition of macromolecules from the tangle conformation to helix and double helix, accompanied by the formation of rigid spatially cross-linked structures.

Nevertheless, the change of conformation of κ-CG-dP macromolecules during synthesis can occur on the surface of nanoparticles spatially isolating them from themselves. The K^+^ and Na^+^ ions present in the macromolecules of κ-CG-dP in the form of counterions of sulfate groups located on the C4 residues of anhydrogalactose links are probably able to bind with tellurium ions present on the surface of growing nanoparticles limiting their growth. In general, the analysis of works devoted to the synthesis of elemental Te^0^NPs allows us to confirm the high probability of obtaining anisotropic particles (nanofibers, nanobelts, nanorods, nanopencils, nanoflowers, nanorice, etc.). Due to its unique helix-chain conformation, Te has a high tendency toward anisotropic growth and formation of one-dimensional (1D) structures due to van der Waals interactions in the hexagonal lattice. At the same time, the shape of such particles, as well as the ratio of their length to width, are regulated by varying the synthesis temperature and using different ratios of reagents, including those involved in the regulation of the pH of the reaction medium, the ionic strength of the solution, and other parameters. In our case, spherical nanoparticles of sufficiently small size were obtained only at a small polysaccharide/Te^2−^ ratio, regardless of the type of polysaccharide. Meanwhile, at the precursor ratio of 10:1, i.e., a larger amount of tellurium in the reaction medium (and, consequently, sodium hydroxide as a hydrolysis product of the tellurium precursor), anisotropic tellurium particles were formed regardless of the type of polysaccharide. This, in turn, allows us to conclude that the choice of synthesis conditions and precursor ratio is of great importance in the development of approaches for the directed production of Te^0^NPs with given nanomorphological characteristics.

#### 3.3.2. Characterization of Polysaccharide-Stabilized Tellurium Nanoparticles in Aqueous Solutions by Dynamic Light Scattering Method

The above TEM and HR-TEM data characterizing first of all the size and polydispersity of Te^0^NPs in Ar-Gal, GM-dP, and κ-CG-dP matrices provide information exclusively about the inorganic component of the obtained nanocomposites, while some practical applications (biology, medicine, optics, analytics, etc.) require information about the complex structure as a whole, which is represented by Te^0^NPs in a polysaccharide matrix. Thus, inorganic nanoparticles, well visualized in TEM or HR-TEM images due to their high contrast, are distributed in the shell of polysaccharide matrices, which are not visualized in images due to the low contrast of the organic component of nanocomposites. Nevertheless, in practical applications, the properties of such hybrid composites are determined not only by the properties of the inorganic core, but also by the “core-shell” structure as a whole, including the nature of the interaction between macromolecules of stabilizing ligands and solvents.

**Figure 5:**
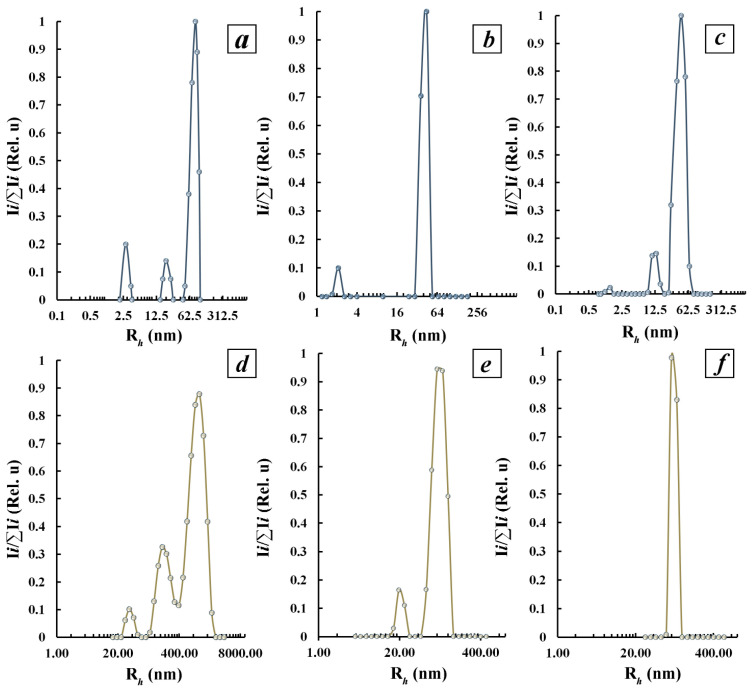
Distribution of hydrodynamic radii (*R_h_*) over the scattering intensity in solutions of Ar-Gal (top row)- and dP-κ-CG-dP (bottom row)-based composites with tellurium content (%) 0—(**a**) and (**d**), respectively, 0.5 (**b**) and 0.8 (**e**), respectively, and 2.6 (**c**) and 3.5 (**f**), respectively.

In this view, the use of “ensemble” methods for obtaining information regarding the size and size distribution of several thousand nanoparticles, for example, by means of dynamic light scattering (DLS) in solution, is a necessary condition for obtaining complete and reliable information on the structure and size characteristics of nanomaterials. According to DLS data, three particle fractions with average hydrodynamic radii (*R_h_*) values of 2.7 nm, 20 nm, and 83 nm, respectively, can be distinguished in the distribution of *R_h_* particles according to their scattering intensity in the aqueous solution of initial Ar-Gal (Figure 5a). Meanwhile, the aqueous solutions of Ar-Gal-stabilized Te^0^NPs in the distribution of particles by their scattering intensity are characterized by a bimodal character of the distribution of fractions (Figure 5b,c) with the identification of both a fast mode corresponding to the diffusive motion of the fraction of small particles and a slow mode corresponding to the diffusive motion of the fraction of large particles. Thus, in the sample of aqueous solution of nanocomposite containing 0.5% Te^0^ two fractions of particles with the value of *R_h_* 2.1 nm and 44.4 nm were detected (Figure 5b). Presumably, the first fast fraction of particles with *R_h_* 1.7–2.1 nm, similar in value to *R_h_* of particles of the fast fraction of the initial Ar-Gal (2.7 nm), corresponds to individual Ar-Gal macromolecules taking the shape of a clew in aqueous medium [41]. Meanwhile, the second fraction of particles with an average *R_h_* of 44.4 nm most likely belongs to intermolecular associations of clew-like Ar-Gal macromolecules with Te^0^NPs formed in them [62].

An increase in the quantitative content of Te^0^ nanoparticles in the nanocomposite up to 2.6% is accompanied by a transition to a three-modal character of nanoparticle distribution and average *R_h_* values of the fast and slow fractions, namely 1.4, 13.2, and 44.4 nm, respectively (Figure 5c). The decrease in the average *R_h_* value of the fast mode to 1.4 nm is probably due to the transition of the conformation of Ar-Gal macromolecules into the form of a densely packed globule, rather than the destruction of Ar-Gal macromolecules during the synthesis of nanocomposites due to the lack of conditions for this. The appearance of another fraction with an average value of 13.2 nm in the *R_h_* distribution of the scattering intensity in this case can also be due to the presence of associates of Ar-Gal macromolecules due to the abundance of hydroxyl groups capable of forming hydrogen bonds. Meanwhile, the slow mode corresponding to the fraction of particles with an average *R_h_* value of 44.4 nm probably also corresponds to intermolecular associations of clew-like Ar-Gal macromolecules with formed in them nanoparticles Te^0^ as in the case of the sample with a smaller amount of tellurium in the composition. The lack of difference in the average *R_h_* values of the particle fraction corresponding to the slow mode in the scattering intensity distribution when changing the tellurium content in the composite is probably due to the aggregation nature of these particles.

Aqueous solutions of κ-CG-dP are also characterized by the presence of three particle fractions with average *R_h_* values of 28.2 nm, 143 nm, and 882 nm (Figure 5d). Most probably, all the selected fractions correspond to particles participating in the formation of intermolecular associates to varying degrees, rather than to individual κ-CG-dP macromolecules. The introduction of 0.8% Te^0^NPs into the composition of κ-CG-dP is accompanied by the transition of the *R_h_* distribution of scattering particles to a bimodal type with the detection of two modes—fast corresponding to the fraction of particles with an average *R_h_* value of 19.8 nm and slow with an average *R_h_* value of 80.5 nm (Figure 5e). In both cases, it is more logical to assume that the separated fractions correspond to associations of κ-CG-dP macromolecules with Te^0^NPs formed in them. Increasing the amount of tellurium in the composition of the composite up to 3.5% is accompanied by changing the *R_h_* distribution of particles to the monomodal type with the isolation of only one mode corresponding to the particles having an average value of *R_h_* 79.6 (Figure 5f). The character of the isolated fraction is probably due to the presence in the aqueous solution of this nanocomposite, which exclusively aggregates κ-CG-dP molecules included in their composite Te^0^NPs.

Composites that contain higher (5.8 and 6.2%) amounts of tellurium in their composition were not investigated by the DLS method due to a significant deviation of their shape from spherical. Composites based on GM-dP were not investigated by this method either due to the known difficulty in interpreting the data of this type of studies to galactomannans because of their pronounced ability to form aggregates in aqueous solutions [63].

#### 3.3.3. Optical Absorption Spectroscopy of Polysaccharide-Stabilized Tellurium Nanoparticles with Different Morphologies

**Figure 6:**
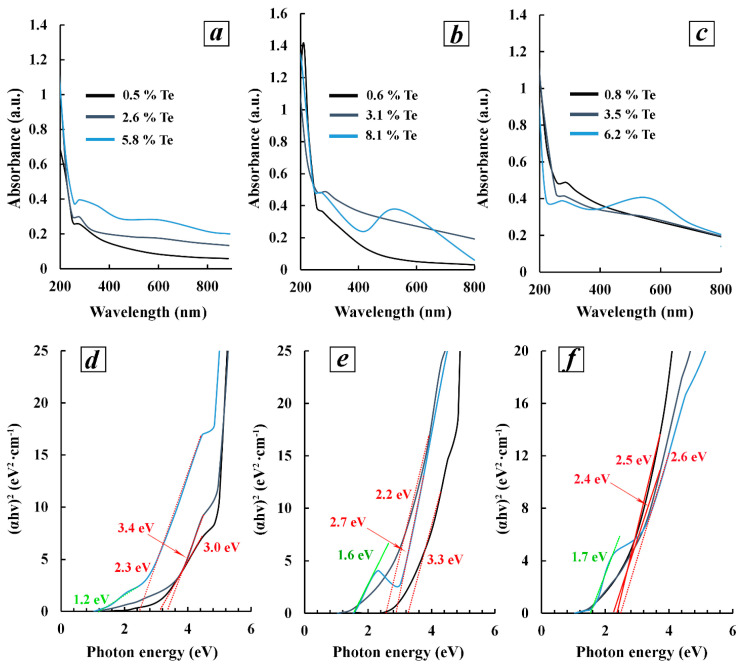
Absorption spectra of 0.1% aqueous solutions of zero-valent tellurium nanocomposites with different inorganic phase contents based on Ar-Gal, dP-GM and κ-CG-dP (**a**), (**b**), (**c**)—respectively; Tauc plots for nanocomposites with different Te weight content on the base of Ar-Gal (**d**), GM-dP (**e**) and κ-CG-dP (**f**).

The optical properties of all the obtained tellurium nanocomposites were investigated by absorption spectroscopy in the UV-visible region of the spectrum. It was found that the spectra of 0.1% aqueous solutions of tellurium nanocomposites based on Ar-Gal, GM-dP, and κ-CG-dP containing 0.5–0.8% inorganic phase have a diffuse character and represent an ascending curve in the high-energy region with a pronounced shoulder in the region of 268–288 nm (Figure 6). Firstly, increasing the quantitative content of tellurium in composites up to 2.6, 3.1, and 3.5%, by an increase in the background absorption for aqueous solutions of composites on all matrices had the most pronounced effect on composites based on Ar-Gal and GM-dP, and secondly, this process was accompanied by the formation of latent absorption in the region of 550–600 nm.

These effects are most likely caused by the increase in the average size of Te under the conditions of increasing its quantitative content in the nanocomposites, as well as the possible beginning of the formation of nanoparticles of anisotropic shape. At the same time, the absorption in the 270–300 nm region (4.6–4.1 eV) is characteristic of Te^0^ nanoparticles [64,65]. Meanwhile, the absorption spectrum of longitudinally oriented Te^0^NPs (nanowires, nanorods, nanobelts, etc.) is characterized by the presence of two bands in the region of 270–300 nm and 530–600 nm [66]. A further increase in the quantitative content of tellurium in the composite by up to 5.8, 8.1, and 6.2% is accompanied by a dramatic change in its appearance, namely, a significant increase in the intensity of absorption in the visible region of the spectrum (533–610 nm) with preservation of absorption in the region of 275–278 nm. In the case of an Ar-Gal-Te^0^NP composite (5.8% Te) containing the largest selenium particles with the maximum length-to-width ratio of 2.1–37.0, the maximum long-wave absorption was observed in the 610 nm region. Meanwhile, in the spectra of anisotropic Te^0^NPs in composites GM-dP-Te^0^NPs (8.1% Te) and κ-CG-dP-Te^0^NPs (6.2% Te), this maximum was located in the region of 538–553 nm. Earlier in [64], we have already described the dependence of the location of the absorption maximum in the spectra of Te^0^NPs on their size. Thus, in this work, with increasing size, the absorption peaks located in the region of 300 nm due to the plasmon-like resonance remain quasi-static, while the peaks due to the Mie-type resonance show red shifts.

Therefore, the next case demonstrates that the character, number and position of maxima in the optical absorption spectra of Te^0^ nanoparticles are directly determined by their size and shape. In our case, the spectra of aqueous solutions of all nanocomposites containing tellurium (0.5–0.8%) are characterized by the presence of a single absorption maximum in the region of 268–286 nm (4.6–4.3 eV) corresponding to inter-zone transitions in Te^0^ nanoparticles (transition from the valence band to the conduction band). The absence of an additional maximum in the region of 450–800 nm is probably due to the formation of Te^0^NPs with a shape similar to that of the spherical one, which is reliably confirmed by TEM data. The increase in the percentage of Te in the nanocomposites is accompanied not only by an increase in the intensity of the absorption maximum in the short-wave region, but also by the appearance of the second broad maximum in the visible region of the spectrum (538–610 nm). Probably, the appearance of the second maximum can be explained by the formation of particles of nonspherical morphology (in particular, Te nanorods), which are characterized by the presence of two absorption maxima in the UV- and visible regions of the spectrum. For all composites, a significant shift of the optical bang gap value towards larger values was observed compared to bulk Te (Figure 6d–f).

It was found that 0.1% aqueous solutions of all obtained nanocomposites were characterized as systems with pronounced colloidal stability, the values of ζ-potential of which lay in the region of negative values and varied in the range of −24–31 mV.

Thus, we evaluated the main characteristics (average size, size distribution, shape) of the Te^0^NPs obtained on the basis of polysaccharide matrices using a set of modern microscopic techniques (which are the “gold” standard for analyzing the morphology of nano-objects) and spectroscopic data. It was found that depending on the synthesis conditions and, first of all, on the ratio of reagents and the type of polysaccharide used, the formed tellurium nanoparticles have different morphology—from close to spherical to rice-like. The obtained data allow us to conclude about the possibility of directed synthesis of tellurium nanoparticles with predictable morphology on the basis of polysaccharide matrices Ar-Gal, GM and κ-CG.

## 4. Discussion

**Figure 7:**
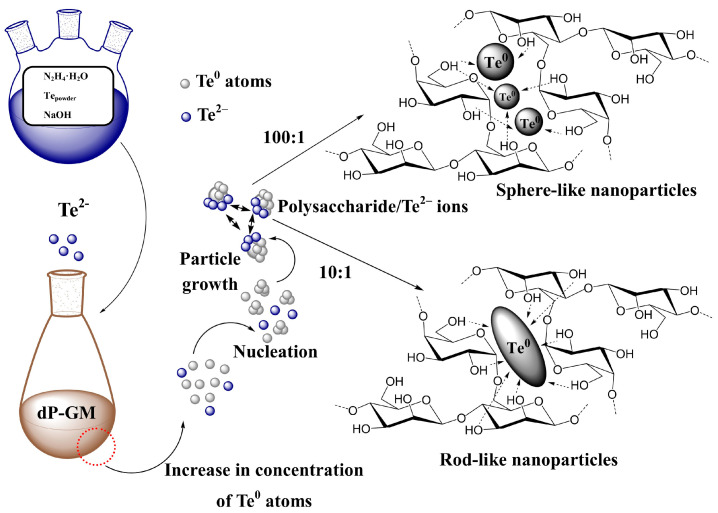
Scheme of the assumed mechanism of formation of Te^0^NPs of different morphology in the matrix of natural galactose-containing polysaccharide dP-GM.

The “hydrazine hydrate-NaOH” base-reducing system which was used for the synthesis of Te^0^NPs was previously actively used not only for the activation of elemental chalcogenes to obtain various functionalized chalcogenorganic compounds [67,68], but also for the synthesis of nanoparticles of elemental chalcogenes—S and Se, including those stabilized by natural polysaccharides [33]. In addition, this system was used to generate highly reactive chalcogenide anions involved in the synthesis of nanoparticles of metal chalcogenides (Ag_2_Se, Bi_2_Se_3_, Bi_2_Te_3_, ZnTe, etc.) [69,70,71]. We also used this system for the synthesis of zero-valent spherical Te^0^NPs stabilized by synthetic copolymer of poly(1-vinyl-1.2.4-triazole) [30]. However, the formation of zero-valent Te^0^NPs in matrices of galactose-containing polysaccharides with investigation of the influence of the type of polysaccharide and synthesis conditions on the nanomorphological characteristics of the obtained nanoparticles is described for the first time in this work. The main advantages of this system are availability and cheapness of initial reagents, as well as environmental friendliness (reaction products are nitrogen and water) and low energy consumption (synthesis of telluride anions is carried out at a temperature not exceeding 70 °C). That is, the use of powder elemental tellurium as the least toxic and most accessible form of tellurium as its source for the synthesis of nanoparticles and highly effective basic-reduction system “hydrazinhydrate-NaOH” capable of activating in fairly mild conditions chemically inert powder tellurium with by-products of the reaction in the form of absolutely environmentally friendly—water and molecular nitrogen is an affordable and safe way to obtain nanoparticles of zero-valent tellurium. Meanwhile, the synthesis in aqueous medium in the presence of macromolecules of non-toxic, accessible and renewable polysaccharides Ar-Gal, GM and κ-CG significantly expands the boundaries of potential application of the nanomaterials obtained and also only strengthens the advantages of the proposed approach to the synthesis of Te^0^NPs over the existing alternative methods of their synthesis, especially in terms of absolute compliance of the approach proposed in this work to the basic principles of “green chemistry”. On the basis of the complex of obtained data on the composition and structure of nanocomposites of elemental tellurium on the basis of natural galactose-containing polysaccharides Ar-Gal, GM-dP and κ-CG-dP, it can be concluded that the most important influence on the morphology of the obtained Te^0^NPs is primarily the ratio of the tellurium precursor and polysaccharide in the reaction medium. It is by varying this ratio that the programmed production of nanoparticles with a shape other than spherical becomes possible. In conditions of a significant excess of polysaccharide macromolecules over the precursors of tellurium in the reaction medium are formed predominantly spherical nanoparticles of tellurium. Meanwhile, the increase of tellurium content is accompanied by the formation of nanoparticles that are predominantly elongated (nanorods, rice-like nanostructures). At the same time, this dependence of the morphology of the obtained Te^0^NPs on the ratio of reagents was observed regardless of the type of polysaccharide used. Schematically, the putative mechanism of formation of Te^0^NPs in the presence of macromolecules of galactose-containing polysaccharides (on the example of galactomannan) is presented in Figure 7.

Rod-shaped particles were formed using branched polysaccharides—Ar-Gal; polysaccharides with a comb structure—GM-dP; and sulfated linear polysaccharides—κ-CG-dP. The difference of nanomorphological characteristics of the obtained Te^0^NPs stabilized from different types of polysaccharides consisted exclusively in their size and degree of polydispersity. Meanwhile, the manifestation of the very tendency to anisotropic growth of nanoparticles along a certain plane of its hexagonal crystal lattice is due to the excess of zero-valent tellurium atoms in the reaction medium capable of oriented attachment along the (001) plane of the crystal lattice of growing tellurium nanocrystallites with the formation of helical chains of covalently linked atoms. Thus, ideal nanocrystallites whose shape is close to rod-shaped can be formed without the use of external influences (pressure, magnetic field, etc.).

## 5. Conclusions

Using the stabilizing capability of natural galactose-containing polysaccharide matrices such as arabinogalactan, galactomannan, and κ-carrageenan, alongside the hydrazine hydrate-alkali base-reducing system to generate highly reactive precursors of tellurium from its least toxic form, water-soluble and aggregate-stable nanocomposites of zero-valent tellurium were obtained and characterized for the first time. It was found that under the specified experimental conditions, the size and shape characteristics of tellurium nanoparticles were primarily influenced by the polysaccharide/Te ratio, as well as the structure and molecular weight of the polysaccharides employed. The discovered features of the most important nanomorphological characteristics of tellurium nanoparticles depending on the synthesis conditions will allow to vary the parameters of the obtained nanoparticles and to obtain nanomaterials with specified properties. The universality of the approach proposed in this work once again successfully demonstrates the advantages of using polysaccharides for the synthesis of nanoparticles due to their availability, renewability and biocompatibility, as well as the ability to synthesize nanomaterials in the maximum non-toxic aqueous environment. The synergetic combination of the complex of properties of polysaccharides used in this work, including their inherent membranotropicity and the complex of properties of nano-particles of zero-valent tellurium allows us to predict the widening of the potential of their possible practical use, including in biomedical fields.

## Data Availability

Data are contained within the article.
